# Association between mean platelet volume and pulmonary embolism: a systematic review and meta-analysis

**DOI:** 10.18632/aging.203205

**Published:** 2021-07-02

**Authors:** Wenyi Lin, Yu Wu, Xuan Lu, Yu Hu

**Affiliations:** 1 Institute of Hematology, Union Hospital, Tongji Medical College, Huazhong University of Science and Technology, Wuhan 430022, China; 2 Department of Ultrasound, Union Hospital, Tongji Medical College, Huazhong University of Science and Technology, Wuhan 430022, China

**Keywords:** mean platelet volume, pulmonary embolism, early death, risk stratification, meta-analysis

## Abstract

Platelet activation plays an important role in the progression of pulmonary embolism (PE). Mean platelet volume (MPV) can serve as a marker of platelet activity in patients with PE. Many studies have reported different results regarding the relationship. Therefore, we aimed to perform a systematic review and meta-analysis to evaluate the relationship between MPV and PE. Two reviewers independently searched relevant articles in databases from inception to April 21, 2021 and identified all studies on MPV and PE as the outcomes of interest. Further, we selected studies meeting the criteria and extracted the data. Of the 2505 publications identified, we included 18 studies after screening. Results showed MPV was significantly higher in patients with PE (0.83 fL, 95% CI: 0.38-1.28, P<0.001) than in controls. The mean difference in MPV between those who died and survivors of PE was 1.23 fL (95% CI: 0.96-1.51, P<0.001). Hence, an increased MPV is associated with PE. MPV could be a useful tool to predict the occurrence and death risk of PE together with other risk factors.

## INTRODUCTION

Pulmonary embolism (PE) is a clinical pathological syndrome caused by various types of emboli (thrombus being the most common). These emboli block the main pulmonary artery or its branches, sometimes causing a life-threatening condition. On the one hand, low-risk PE can be asymptomatic or identified incidentally, with a mortality rate of less than 1% [1]. On the other hand, high-risk PE, which presents as shock or persistent hypotension, is a life-threatening condition associated with high mortality and morbidity, with an overall mortality rate exceeding 10% within 30 days [2–5]. Early assessment of PE risk plays an important role in guiding clinical treatment and reducing patient mortality. In recent years, computed tomography pulmonary angiography (CTPA) and magnetic resonance imaging (MRI) have been widely used to diagnose patients with PE. However, these examinations are relatively costly and therefore less feasible and of limited use in primary hospitals.

Recently, many studies have reported that in the early stage of PE, various visible and invisible components that produce hypercoagulable substances are found in the patients’ blood. This detection can sensitively reflect PE. Mean platelet volume (MPV), a measurement of platelet size, is a widely used indicator assessing platelet function and activity [6, 7]. Large-sized platelets contain more dense granules, generate more vasoactive and prothrombotic factors (e.g., thromboxane A2, serotonin, and adenosine triphosphate), secrete more membrane receptors (e.g., P-selectin and glycoprotein IIb/IIIa), and aggregate more rapidly [7–9]. A high MPV indicates a rapid hemostatic reaction and a higher thrombotic propensity [10, 11]. Therefore, MPV can be used as a predictor of the occurrence and poor prognosis of thrombotic diseases.

A meta-analysis on thrombosis and MPV published in 2017 reported that the onset of PE had no significant effect on the standardized mean difference (MD) of MPV between patients with PE and controls [12]. However, large sample studies on MPV and PE were subsequently conducted [13, 14], and they showed a significant increase in MPV among patients with new PE onset and recurrent PE. A meta-analysis published in 2020 demonstrated an increased MPV associated with PE but not assessed the use of MPV as an indicator for the risk prediction and risk stratification of PE [15]. In view of this, we conducted a systematic review and meta-analysis with an aim to further evaluate the relationship between MPV and PE.

## MATERIALS AND METHODS

The meta-analysis was carried out according to PRISMA guidelines [16].

### Search strategy

Two reviewers (WY Lin and X Lu) independently searched relevant studies in the PubMed, Web of Science, SCOPUS, and OVID (including Embase and Medline) databases from their inception to April 21, 2021, using the search terms (“pulmonary embolism” OR “PE” OR “pulmonary thromboembolism” OR “PTE” OR “lung embolism”) AND (“mean platelet volume” OR “MPV” OR “platelet indices” OR “platelet parameters”). In addition, reference lists of the relevant studies were reviewed to identify eligible studies.

### Selection criteria

We included studies according to the following criteria: 1) a cohort or case–control design; 2) a clear diagnosis of PE by CT, CTPA, or MRI; 3) reporting the mean and standard deviation (SD) of MPV for patients with PE and controls or for survivor and death groups, or reporting frequencies for subjects in high-/low-MPV groups, numbers of patients and subjects or controls, or odds ratios (OR)/risk ratios (RR)/hazard ratios (HR) with 95% confidence intervals (CI); 4) full text available.

Studies were excluded if they were duplicates, non-English, reviews, letters, case reports, books, meeting abstracts (with no data of the outcome), nonhuman, did not provide data of MPV, did not include an outcome of PE or early death, and did not include a control group. Further, studies that reported MPV with median values and studies in which participants had concurrent diseases that influenced the outcomes of interest were also excluded. For the same study with several publications, we chose the most recent data or the paper with the longest follow-up, or we contacted the primary/corresponding author to confirm differences between these studies, when necessary.

All identified studies were screened initially by their title or abstract, and then by their full text. Two reviewers (WY Lin and X Lu) independently evaluated each study, and inconsistent articles were checked by the corresponding author (Y Hu).

### Data extraction

The reviewers then independently extracted information of the included studies in a Microsoft Excel spreadsheet. The study information included details of the authors, year, region, study design, data source, data collection period, follow-up duration, diagnostic criteria, standards of risk stratification, inclusion criteria of participants, and number of cases and controls or survivors and non-survivors, and number of high-/medium-/low-risk cases. In addition, we recorded statistical information of the number of events at follow-up, mean and SD of MPV, OR, RR, HR with 95% CI, the number and type of covariates considered in analyses, sample size, average patient age, proportion of males, and PE detection methods (including whether samples were collected with ethylenediaminetetraacetic acid (EDTA), time before measurement, and details of testing analyzers). Finally, we collected clinical information on the percentage of smokers, percentage of subjects with diabetes mellitus and hypertension, whether participants received anticoagulation therapy, history of deep vein thrombosis (DVT), and history of cardiovascular diseases or other diseases influencing the outcome of interest. Controversial information was re-reviewed by consensus or judged the corresponding author (Y Hu). Missing data were obtained by consulting the primary authors of the studies.

### Risk of bias assessment

Risk of bias for included studies was evaluated independently by the two reviewers with the Newcastle–Ottawa Scale (NOS) [17]. This scale assesses the selection of the study participants, the comparability of patients and controls in case–control studies and exposed and nonexposed participants in cohort studies, and the ascertainment of exposure or outcome. Disagreement was resolved by consensus or judged by the corresponding author.

### Statistical analysis

For continuous outcomes, the mean differences (MD) in MPV between patients and controls or survivors and non-survivors in each study was evaluated; then, weighted mean differences (WMD) and 95% CI were pooled. For dichotomous outcomes, OR, RR, and HR with 95% CI were calculated or obtained directly from the study data on PE or early death among high- versus low-MPV participants. Heterogeneity among studies was measured with the chi-square test, whereas the degree of heterogeneity was calculated with I-square (I^2^) statistics. When heterogeneity was significant (P < 0.1 or I^2^ > 50%), a random effects model was used; otherwise, a fixed effects model was used [18, 19].

The sources of heterogeneity were analyzed with a meta-regression model and subgroup analysis by region, testing time, analyzer, smoking, diabetes mellitus, type of disease, and the score of NOS. Sensitivity analysis was applied to increase the credibility and robustness of the final results, mainly by deleting each of the included studies, one at a time, and then analyzing the pooling effect and heterogeneity of the remaining papers. Publication bias was evaluated by constructing a funnel plot, performing Egger’s test, and calculating Begg-Mazumdar Kendall’s tau value [20, 21]. All statistical tests were two-sided, and P <0.05 denoted significance, in addition to the heterogeneity test that was set at 0.1. All analyses mentioned above were performed in STATA statistical software, version 14.0.

## RESULTS

**Figure 1:**
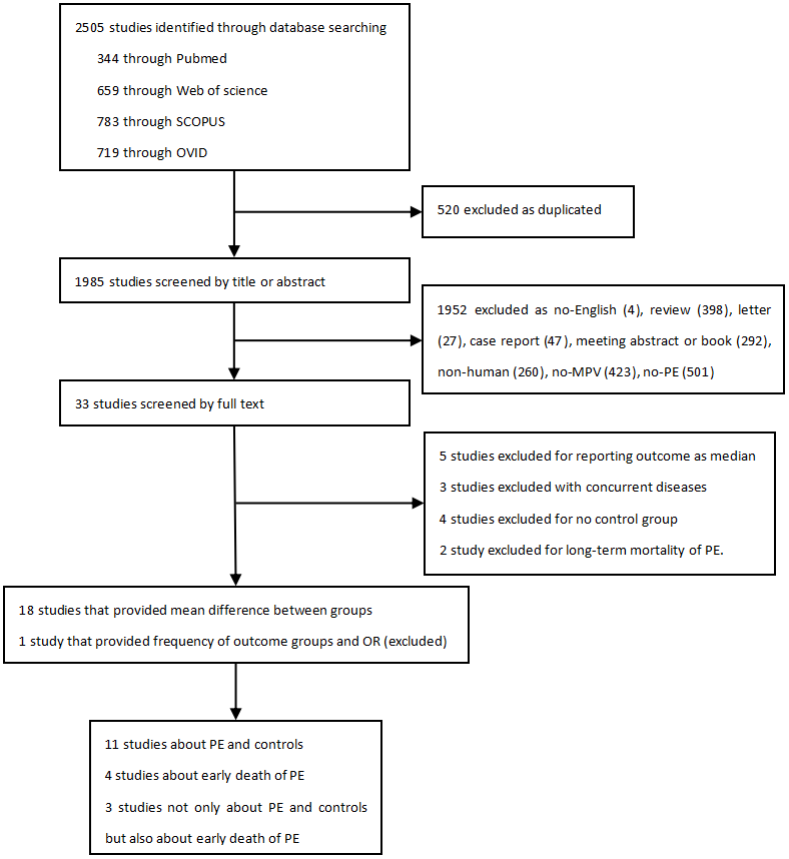
Flow chart of study selection.

A total of 2505 relevant publications were identified, and the screening process was done as shown in Figure 1. Finally, 18 studies met the selection criteria and were included in the meta-analysis, with 14 studies on PE [3, 14, 22–33] and 7 studies on early death of patients due to PE [3, 13, 24, 26, 34–36].

All included studies were case–control studies, and they included 2674 patients and 1192 controls in total. Most studies were conducted in Turkey, except one study in China, one in Poland, one in Italy, one in Iran and one in Egypt. The mean age of all subjects ranged from 40 to 74 years, and the percentage of males, smokers, and patients with diabetes ranged from 40% to 59%, 19% to 54%, and 8% to 30%, respectively. Other characteristics of each study are shown in Table 1 and Supplementary Table 1.

**Table 1:** Characteristics of included studies.

**Author**	**Year**	**Region**	**Size**	**Age (year)**	**Male (%)**	**EDTA**	**Testing time**	**Analyzer**	**Smoker (%)**	**Diabetics (%)**	**Type**	**NOS**
**Studies about PE**												
Sentürk, A.	2017	Turkey	480	67.7	45	Y	NA	Sysmex	19	9	N	6
Huang, J.	2015	China	145	58.5	52	Y	NA	Sysmex	NA	EX	N	7
Sunnetcioglu, A.	2014	Turkey	120	57.4	44	Y	30min	Beckman	NA	NA	NA	7
Talay, F.	2014	Turkey	315	51.5	59	Y	60min	Sysmex	37	16	S	6
Guna, E	2013	Turkey	113	57.0	59	Y	60min	Sysmex	54	NA	NA	8
Hilal, E.	2013	Turkey	371	61.6	52	NA	NA	Beckman	NA	18	NA	7
Varol, E.	2011	Turkey	177	59.4	49	Y	30min	Beckman	21	NA	S	7
Kostrubiec, M.	2010	Poland	292	64.3	40	Y	30min	Advia	NA	NA	NA	6
Sevuk, Utkan	2015	Turkey	100	39.5	51	Y	NA	Sysmex	42	EX	A	7
Icli, Atilla	2015	Turkey	196	54.6	48	Y	120min	Beckman	19	8	A	7
In, E.	2015	Turkey	187	57.5	53	Y	NA	Advia	NA	NA	N	8
Moharamzadeh, P.	2019	Iran	173	60.1	50	NA	NA	NA	NA	NA	NA	6
Abd, E.	2019	Egypt	70	48.6	40	NA	NA	NA	29	16	S	5
Çevik, I.	2014	Turkey	128	64.7	NA	NA	NA	Beckman	NA	NA	NA	5
**Studies about early death**												
In, E.	2015	Turkey	108	58.1	54	Y	NA	Advia	NA	NA	N	6
Hilal, E.	2013	Turkey	209	62.4	51	NA	NA	Beckman	NA	18	NA	7
Kostrubiec, M.	2010	Poland	192	64.0	41	Y	30min	Advia	NA	NA	NA	6
Araz, O.	2017	Turkey	440	61.0	46	Y	15min	Beckman	NA	NA	NA	6
Akgullu, C.	2015	Turkey	206	61.8	47	NA	NA	NA	43	30	S	7
Ertem, A	2016	Turkey	264	67.6	46	Y	60min	NA	NA	NA	S	7
Bozkus, F.	2015	Turkey	89	59.1	55	Y	NA	Cell-dyn	NA	13	NA	6

Y: blood samples containing EDTA; EX: subjects with diabetics were excluded; A: all PE patients were combined with DVT; S: some PE patients were combined with DVT; N: No PE patients were combined with DVT; NA: not available.

### Pooling PE events as outcome

**Figure 2:**
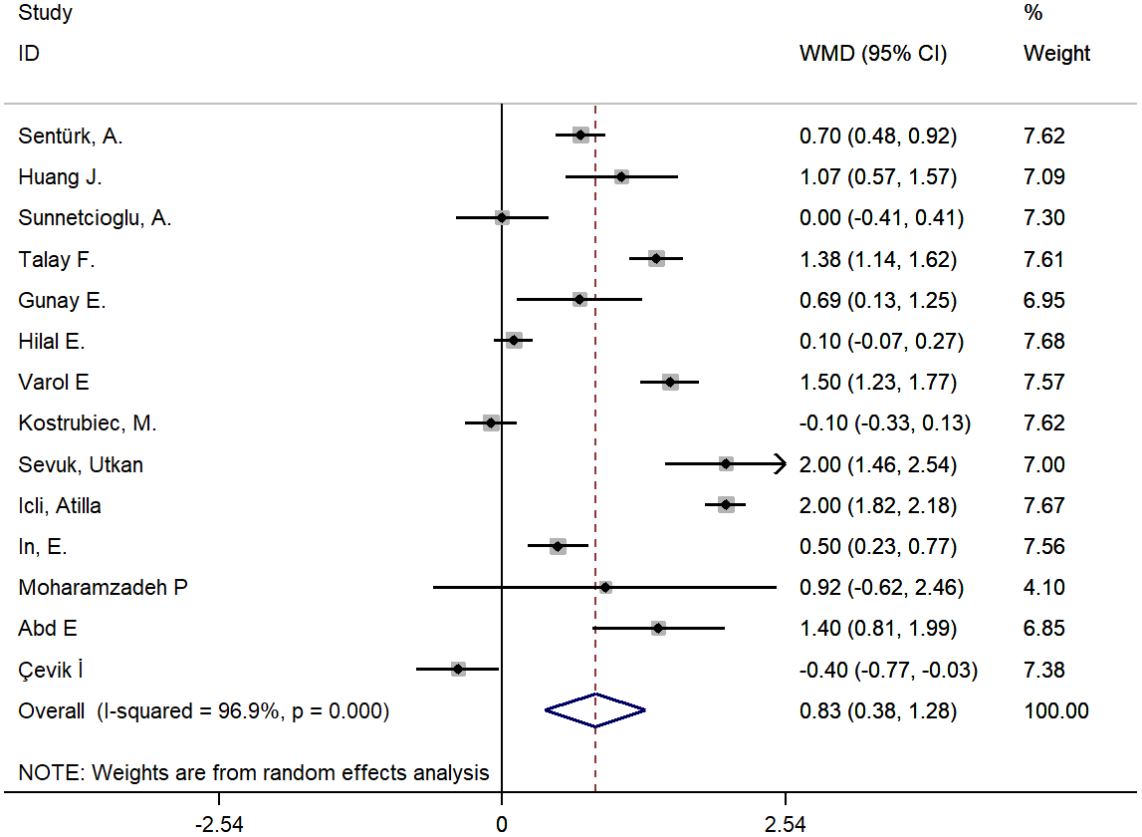
Pooling of weighted mean difference (WMD) for studies about PE.

**Figure 3:**
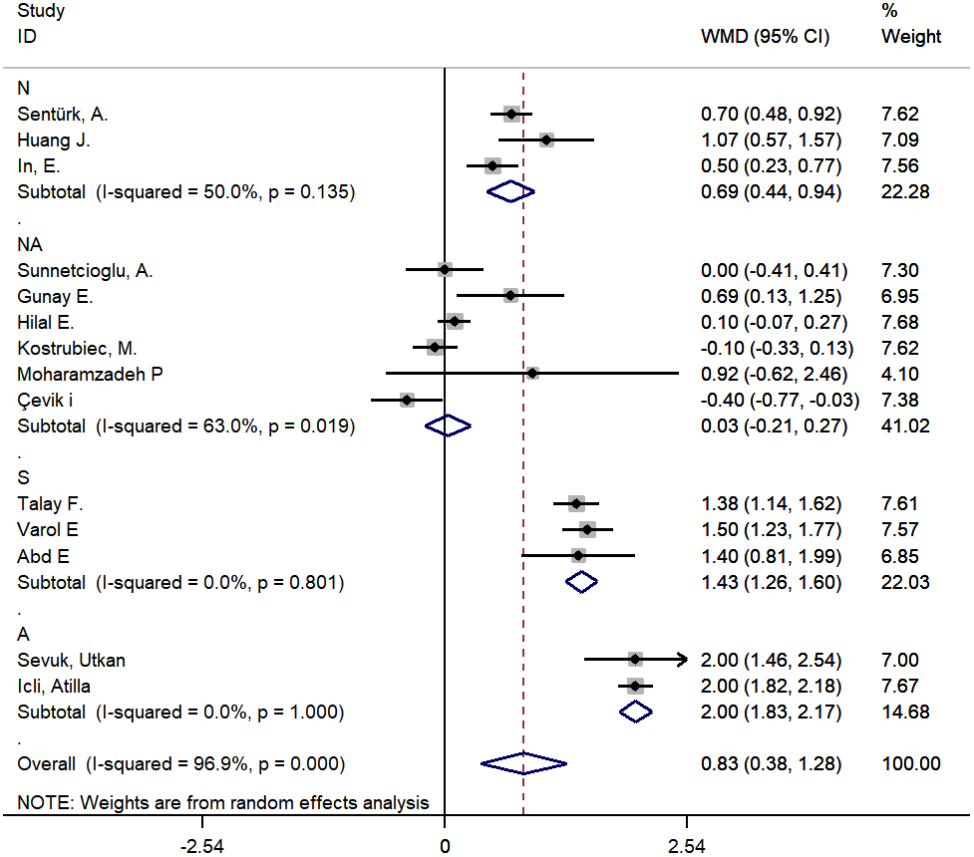
**The subgroup analysis by type of disease for studies about PE.** A: all PE patients were combined with DVT; S: some PE patients were combined with DVT; N: No PE patients were combined with DVT; NA: not available.

**Figure 4:**
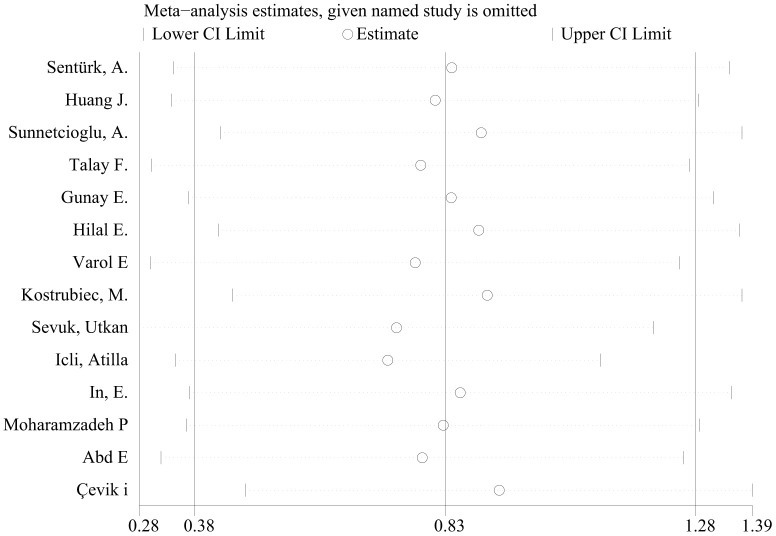
The sensitivity analysis for studies about PE.

In the meta-analysis of studies on PE, a larger MPV was found in cases versus controls with a WMD of 0.83 fL (95% CI: 0.38-1.28, P<0.001). However, the heterogeneity was substantial, and hence, a random effects model was used (chi-square = 418.81, df =13, I^2^ =96.9%, P<0.001) (Figure 2). A meta-regression analysis was conducted, and the results showed that age, smoking and type of disease might be sources of heterogeneity (coefficient=-0.076, P=0.007; coefficient=-1.17, P=0.001; coefficient=1.34, P<0.001, respectively). The subgroup analysis showed disease type might be a source of heterogeneity and the pooled MD of MPV in the subgroup of PE patients with DVT was more significant than that without DVT (Figure 3 and Supplementary Table 2). The sensitivity analysis suggested that the finding regarding the role of MPV in PE was robust because pooling MPV and the heterogeneity did not vary substantially, no matter which study was removed (Figure 4 and Supplementary Table 3). The funnel plot presented symmetry (Supplementary Figure 1), and Begg’s test and Egger’s test did not reveal evidence of publication bias (Begg’s test, P=0.584; Egger’s test, P=0.145).

### Pooling early death as outcome

**Figure 5:**
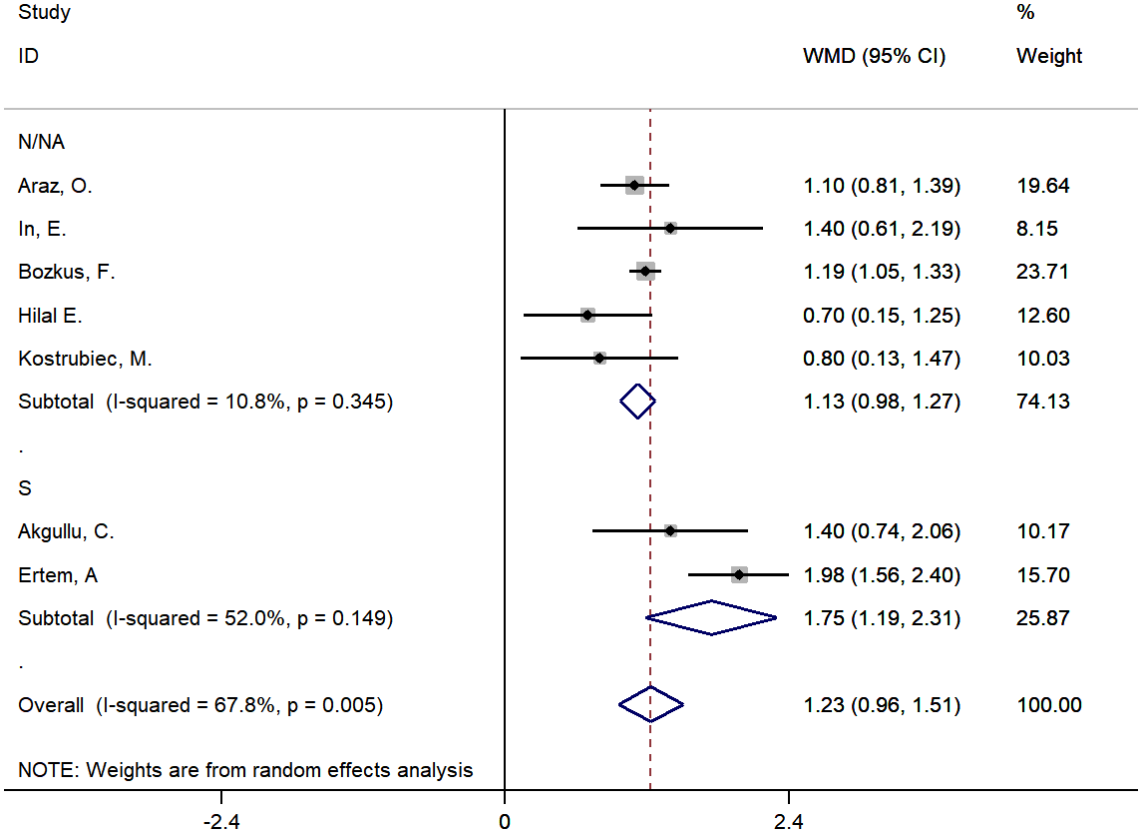
**The subgroup analysis by type of disease for studies about early death of PE.** S: PE patients were combined with DVT; N/NA: No PE patients were combined with DVT or not available.

**Figure 6:**
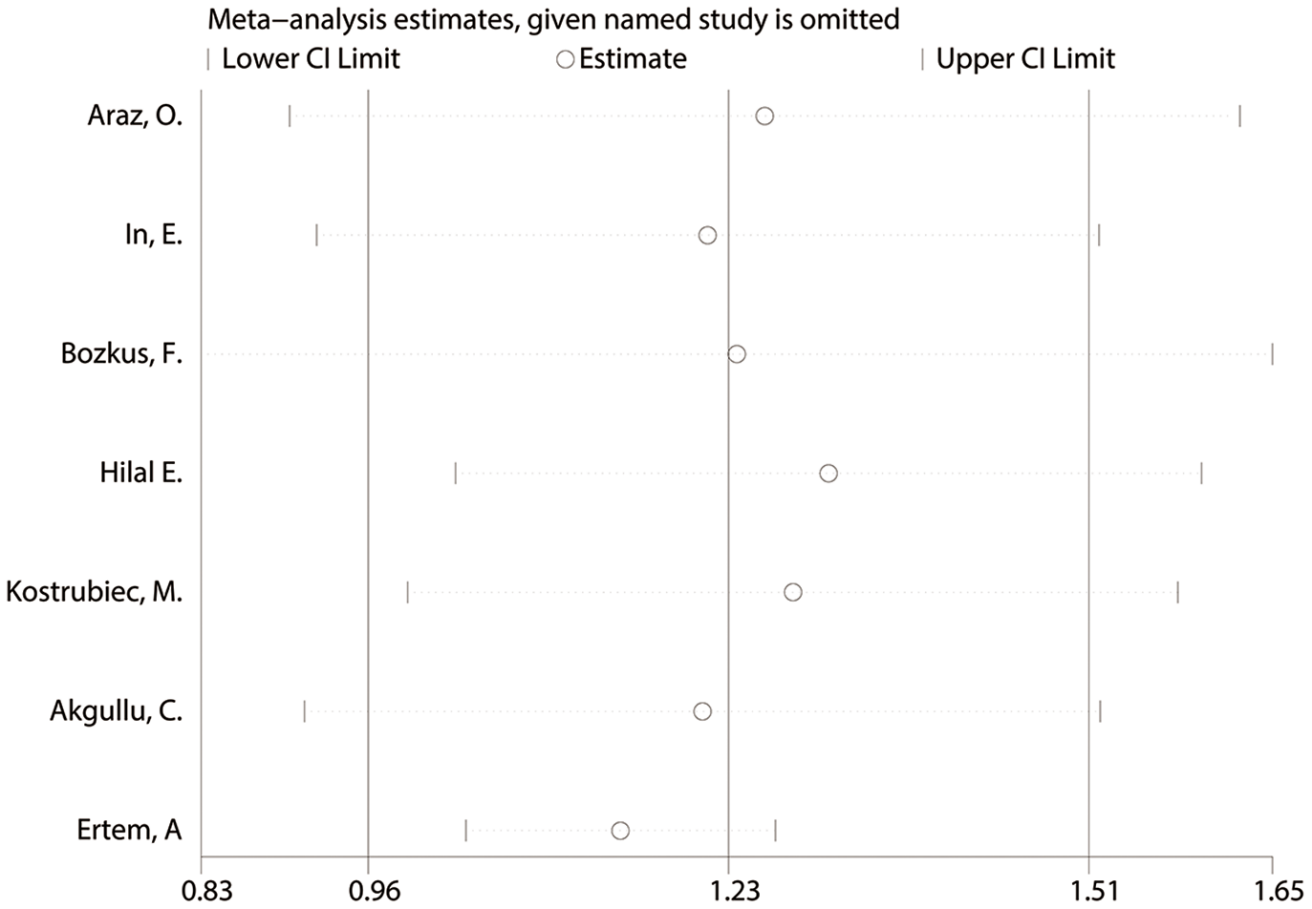
The sensitivity analysis for studies about early death of PE.

Characteristics of each study on early death of PE are shown in Table 1 and Supplementary Table 4. The pooled MD of MPV between those who died and survivors was approximately 1.23 fL (95% CI: 0.96-1.51, P<0.001). A random effects model was used for large heterogeneity (chi-square=18.61, df=6, I^2^=67.8%, P=0.005). The subgroup analysis found that disease type might be a source of heterogeneity, which was significantly declined in the subgroup of PE patients with DVT. (Figure 5 and Supplementary Table 5). The sensitivity analysis indicated a stable pooling result (Figure 6 and Supplementary Table 6). However, the heterogeneity lowered to 0.6% after removing the study by Ertem AG, et al. [31], in which more than half of the patients had DVT as a complication. In addition, the funnel plot exhibited significant asymmetry, reflecting the existence of publication bias (Supplementary Figure 2).

## DISCUSSION

We conducted a systematic review and meta-analysis of 18 studies that analyzed the association between MPV and PE. Our results indicated that MPV was significantly higher, approximately 0.83 fL, in patients with PE than in controls, and MPV was 1.23 fL higher in those who died than in survivors of PE.

Some meta-analyses have been performed to assess the relationship between MPV and arterial thrombosis such as coronary artery disease and ischemic heart disease [37–39]. The results showed that patients with arterial thrombosis had higher MPVs than controls. Moreover, the study by Kovacs S, et al. [12] had reached a consensus on the association between MPV and venous thromboembolism (VTE), which demonstrated that the pooled MD of MPV was 0.69 fL between patients and controls. Additionally, the meta-analysis by Febra C, et al. [15] had described an increased MPV associated with PE. However, the study by Febra C did not include sufficient research and not find a significant source of high heterogeneity for the pooled result of MPV. Moreover, some studies showed differences in MPV between died PE patients and survivors [3, 13, 24, 26, 34–36], but there was no meta-analysis evaluating the predictive capacity of MPV for the early death of PE. Our meta-analysis pooled various results of relevant studies to address this issue and fully assess the role of MPV in the risk prediction of PE and the prophylaxis of early death due to PE.

### Relationship between MPV and PE

PE is a pathological condition that recurs frequently and is related to increased death and considerable healthcare costs [2]. Pulmonary thromboembolism (PTE), always along with DVT, is the most common type of PE. Platelet activation is the first step of thrombosis, including size change, adhesion, aggregation, and release of a large number of active factors. MPV is a simple marker of platelet activation. Large-sized platelets are thus more reactive, aggregate more rapidly, lead to reduced bleeding time, and have a higher thrombotic propensity [7–11]. Therefore, MPV might be higher in patients with PE and could be an easy indicator for risk assessment and prediction of early death, which was consistent with the results of our meta-analysis. However, we couldn’t demonstrate the utility of MPV in the prediction of PE recurrence, as the study by Araz O, et al. [13] described, for the relevant literature too few to be included. Though uncertain, we thought the hypothesis would be proved in the future.

The pooled results presented heterogeneity to different degrees. The following factors may be sources of heterogeneity: basic features of the study population (age, smoking), disease type (PE with or without DVT), and publication bias. The relation between age and MPV was controversial, with some studies showing no difference in MPV among different age groups, while others revealing MPV increasing with age [40–43]. The mean age difference between studies might have caused the substantial heterogeneity. The negative effect of age for MPV in our meta-analysis was probably due to other confounding factors or due to false negatives for the small number of studies in the meta-regression analysis. Studies indicated a higher MPV in smokers than in nonsmokers, and smoking cessation has been shown to decrease the value of MPV [44, 45]. In our meta-analysis, the proportion of smokers in each study positively correlated to MPV. Therefore, smoking might be a confounding factor influencing the real association of MPV with PE and may have resulted in the heterogeneity.

Notably, in the study by Ertem AG [35], the MD of MPV between patients with PE and those without PE was higher than that in other studies. In addition, the heterogeneity was considerably lower after removing this study. The most likely reason was that more than half of the patients had DVT as a complication. The study by Kovacs S [12] showed MPV was approximately 0.66 fL higher in patients with DVT than in controls. Patients with PE along with DVT might present greater MPV than those without DVT. Therefore, studies containing more cases with DVT might have a more significant pooled difference in MPV than those including fewer subjects with DVT, which was in accordance with the results of our subgroup analysis by the disease type. Thus, the study by Ertem AG [35] yielded markedly different results and resulted in heterogeneity.

Additionally, some studies showed that there was no significant difference in MPV between patients and controls among the included studies [3, 25, 26, 31]. This may be due to the different effects of MPV in patients with different levels of risk for PE. Studies by Varol E [28] and Gunay E [22] presented huge differences in MPV of patients with high, medium, and low risk, while studies by Kostrubiec M [26], Hilal E [3], and Ates H [46] showed no differences in MPV. It was unknown whether the differences in MPV between patients with different risk and controls were significant or not. It is possible that studies including more patients with a high risk presented a larger difference in MPV between patients and controls, while studies including more patients with a low risk exhibited no difference in MPV. However, not all studies conducted risk stratification for patients and described the proportion of patients with different risk levels. Therefore, we thought the inclusion of patients at different risk levels in studies was another source of heterogeneity. Furthermore, not all studies have identified the diagnose of PTE. PTE is only the main part of PE, and there are still PE types that are not PTE. Whether MPV is also related to this part of PE patients is still unknown and need more studies to certificate. This may be another reason for some studies finding no significant difference in MPV between PE patients and controls.

### Strengths and limitations

Compared to the previous meta-analysis by Febra C [15], there are many other strengths in our study. We identified studies by an in-depth search in 4 databases. Two additional study [14, 30] on PE and 4 additional studies [13, 34–36] on early death due to PE were included. The study by Farokhi M [47] on PE was excluded for abstract. We not only described the association between MPV and PE but also assessed the predictive effect of MPV on early death among patients with PE. This study is the first meta-analysis to demonstrate an increased MPV in PE patients with early death. In addition, we explained the sources of heterogeneity in detail according to subgroup, meta-regression, and sensitivity analyses. Furthermore, the two reviewers independently screened the studies according to the criteria and extracted the data. A total of 18 studies were included in the review, with the number of subjects reaching 2674 cases and 1192 controls.

However, this meta-analysis also has a few limitations. First, all included studies were case–control studies, which could not help assess the predictive or prognostic role of an increased MPV in PE. Second, there were many confounding factors influencing MPV, which were not described or not considered in the included studies. Therefore, sources of high heterogeneity could not be identified completely. It remains uncertain whether the role of MPV in PE is direct or indirect through mediation by other factors. Thus, the independent effect of MPV on PE might be overestimated. In addition, further research should be carried out to address the existing publication bias.

### Clinical application and future research

Testing for MPV is cheap and easy in clinical practice. The results of our meta-analysis suggest that MPV is a useful indicator to predict the risk of the occurrence of PE and its related death. But the pooling MPV presented high heterogeneity. Therefore, MPV could be used as a tool to help for the diagnosis and risk score of PE together with many other identified risk factors (Supplementary Table 7). Moreover, MPV could be used for risk stratification of PE and added to the current guidelines [48] to improve the risk estimation accuracy and increase the survival rate. Additionally, MPV could be used as a marker to estimate the effect of treatment, such as anticoagulant and thrombolytic therapies [13, 49, 50].

Further studies should be conducted to support our findings and to examine the clinical utility of MPV testing among patients with PE. Future large-scale cohort studies that include complete information on all known risk factors for VTE should be conducted, and these should consider all confounding factors for MPV, such as age, sex, smoking, diabetes mellitus, EDTA use, type of analyzers used, and testing time. Furthermore, the subjects included in the future studies should be classified by risk stratification to compare the different effects of MPV between patients with different risk levels and controls.

## CONCLUSIONS

There was a positive association between MPV and PE. MPV was significantly larger in patients with PE than in controls and in those who died than in survivors of PE. These findings indicate that MPV could be a useful marker for risk prediction and risk stratification in patients with PE together with other risk indicators.

## Supplementary Material

Supplementary Figures

Supplementary Tables
