# Supplementary material for: Association between mean platelet volume and pulmonary embolism: a systematic review and meta-analysis
Source: Aging (Albany NY). 2021 Jul 2;13(13):17253–73. doi: 10.18632/aging.203205 (PMC8312463; doi:10.18632/aging.203205)
Supplement: Supplementary Figures [file aging-13-203205-s001.pdf]

SUPPLEMENTARY FIGURES

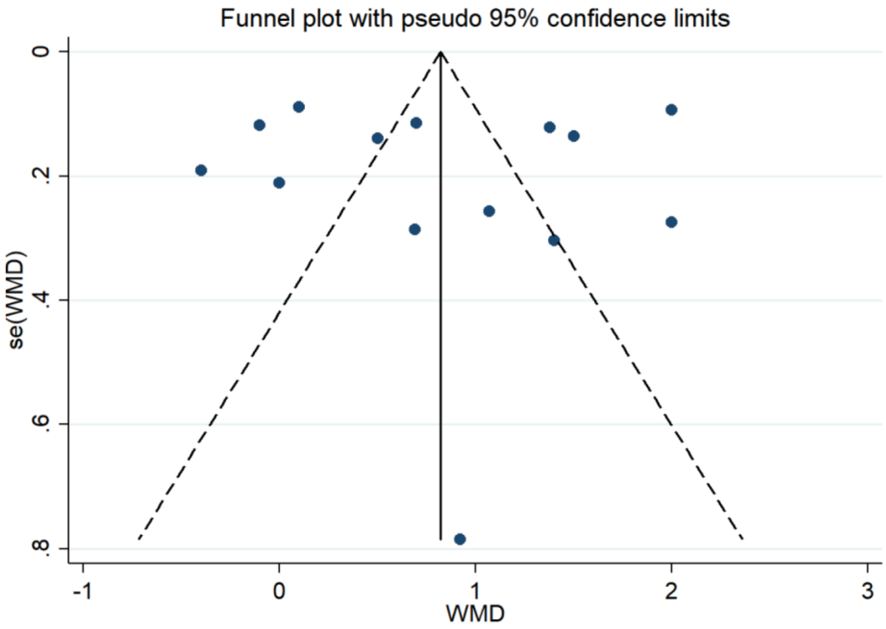

Supplementary Figure 1. The funnel plot of studies of PE.

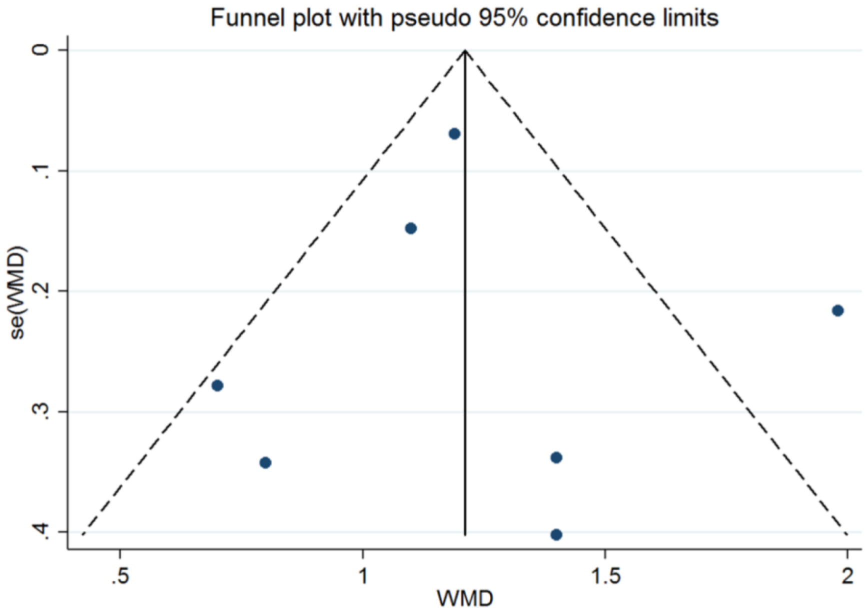

Supplementary Figure 2. The funnel plot of studies of early death of PE.
