# Supplementary material for: Association between mean platelet volume and pulmonary embolism: a systematic review and meta-analysis
Source: Aging (Albany NY). 2021 Jul 2;13(13):17253–73. doi: 10.18632/aging.203205 (PMC8312463; doi:10.18632/aging.203205)
Supplement: Supplementary Tables [file aging-13-203205-s002.pdf]

SUPPLEMENTARY TABLES

Supplementary Table 1. Data of MPV (fL) for each study about PE.

Author	year	ne	emean	esd	nc	cmean	csd
Sentürk, A.	2017	325	9.8	1.7	155	9.1	0.8
Huang, J.	2015	70	9.91	1.4	75	8.84	1.68
Sunnetcioglu, A.	2014	67	8.4	1.2	53	8.4	1.1
Talay, F.	2014	150	9.42	1.22	165	8.04	0.89
Gunay, E.	2014	63	10.92	1.37	50	10.23	1.61
Hilal, E.	2013	209	8	1.1	162	7.9	0.59
Varol, E.	2011	107	9.6	1	70	8.1	0.8
Kostrubiec, M.	2010	192	10	1.2	100	10.1	0.8
In, E.	2015	108	8.9	1.1	79	8.4	0.8
Sevuk, Utkan	2015	50	10	1.6	50	8	1.1
Icli, Atilla	2015	98	9.9	0.6	98	7.9	0.7
Moharamzadeh, P.	2019	125	10.38	8.59	48	9.46	1.11
Abd, E.	2019	50	8.9	1	20	7.5	1.2
Çevik, I.	2014	61	9.73	1.19	67	10.13	0.94

ne/nc: the number of subjects in case or control group.
emean/cmean: the mean value of MPV in case or control group.
esd/csd: the standard deviation of MPV in case or control group.

Supplementary Table 2. The subgroup analysis for studies about PE.

PE	No.of studies	WMD (95%CI)	Heterogeneity	
			I^2	P-value
Region				
W	11	0.76 (0.25-1.27)	97.6%	<0.001
NW	3	1.19 (0.82,1.56)	0.0%	0.665
Testing time				
≤2h	6	0.92 (0.16-1.68)	97.9%	<0.001
NA	8	0.74 (0.29-1.18)	92.0%	<0.001
Analyzer				
Sysmex	5	1.16 (0.71-1.60)	86.8%	<0.001
Beckman	5	0.65 (-0.33-1.62)	98.7%	<0.001
Advia	2	0.20 (-0.39-0.78)	90.8%	0.001
NA	2	1.34 (0.78-1.89)	0.0%	0.568
Smoker				
Y	7	1.39 (0.95-1.82)	93.3%	<0.001
NA	7	0.19 (-0.11-0.49)	82.1%	<0.001
Diabetics				
Y	5	1.11 (0.32-1.90)	98.3%	<0.001
EX	2	1.53 (0.62-2.44)	83.7%	0.013
NA	7	0.41 (-0.17-0.98)	94.4%	<0.001
Type of disease				
S	3	1.43 (1.26-1.60)	0.0%	0.801
N	3	0.69 (0.44-0.94)	50.0%	0.135
NA	6	0.03 (-0.21-0.28)	63.0%	0.019
A	2	2.00 (1.83-2.17)	0.00%	1.000
NOS				
5	2	0.49 (-1.28-2.25)	96.0%	<0.001
6	4	0.69 (-0.06-1.45)	96.1%	<0.001
7	6	1.11 (0.28-1.94)	98.1%	<0.001
8	2	0.54 (0.29-0.78)	0.0%	0.55

W/NW: white or non-white country; Y: subjects contain smokers (or diabetics); EX: subjects with diabetics were excluded; A: all PE patients were combined with DVT; S: some PE patients were combined with DVT; N: No PE patients were combined with DVT; NA: not available.

Supplementary Table 3. The sensitivity analysis for studies about PE: by deleting one of the studies and then analyzing the pooling effect and heterogeneity of the remaining papers.

Author	No.of subjects	WMD (95%CI)	Heterogeneity	
			I^2	P-value
Sentürk, A.	2387	0.84 (0.34-1.35)	97.1%	<0.001
Huang, J.	2722	0.81 (0.34-1.29)	97.1%	<0.001
Sunnetcioglu, A.	2747	0.90 (0.42-1.37)	97.0%	<0.001
Talay, F.	2552	0.79 (0.30-1.27)	97.0%	<0.001
Gunay, E.	2754	0.84 (0.37-1.32)	97.1%	<0.001
Hilal, E.	2496	0.89 (0.41-1.36)	96.5%	<0.001
Varol, E.	2690	0.78 (0.30-1.26)	96.9%	<0.001
Kostrubiec, M.	2575	0.91 (0.45-1.37)	96.6%	<0.001
Sevuk, Utkan	2767	0.74 (0.28-1.21)	97.0%	<0.001
Icli, Atilla	2680	0.73 (0.34-1.11)	94.7%	<0.001
In, E.	2671	0.86 (0.37-1.35)	97.1%	<0.001
Moharamzadeh, P.	2694	0.83 (0.36-1.29)	97.1%	<0.001
Abd, E.	2797	0.79 (0.32-1.26)	97.1%	<0.001
Çevik, I.	2739	0.93 (0.47-1.39)	96.8%	<0.001

Supplementary Table 4. Data of MPV (fL) for each study about early death of PE.

Author	year	ne	emean	esd	nc	cmean	csd
Araz, O.	2017	73	8.8	1.2	367	7.7	0.9
In, E.	2015	11	10.2	1.3	97	8.8	0.9
Bozkus, F.	2015	11	8.78	0.19	78	7.59	0.35
Hilal, E.	2013	17	8.6	1.1	192	7.9	1.1
Kostrubiec, M.	2010	18	10.7	1.4	174	9.9	1.2
Akgullu, C.	2015	30	10.2	1.81	1.76	8.8	0.96
Ertem, A	2016	34	10.64	1.14	230	8.66	1.41

ne/nc: the number of subjects in death or survivor group.
emean/cmean: the mean value of MPV in death or survivor group.
esd/csd: the standard deviation of MPV in death or survivor group.

Supplementary Table 5. The subgroup analysis for studies about early death of PE.

Early death	No.of studies	WMD (95%CI)	Heterogeneity	
			I^2	P-value
Testing time				
≤2h	3	1.32 (0.65-1.98)	85.5%	0.001
NA	4	1.16 (0.94-1.38)	19.2%	0.294
Diabetics				
Y	3	1.12 (0.81-1.42)	41.1%	0.183
NA	4	1.34 (0.80-1.87)	78.3%	0.003
Type of disease				
S	2	1.75 (1.19-2.31)	52.0%	0.149
N/NA	5	1.13 (0.98-1.27)	10.8%	0.345
NOS				
6	4	1.16 (1.05-1.29)	0.00%	0.615
7	3	1.37 (0.57-2.17)	84.9%	0.001

Y: subjects contain diabetics; S: some PE patients were combined with DVT; N/NA: No PE patients were combined with DVT or not available.

Supplementary Table 6. The sensitivity analysis for studies about early death of PE: by deleting one of the studies and then analyzing the pooling effect and heterogeneity of the remaining papers.

Author	No.of patients	WMD (95%CI)	Heterogeneity	
			I^2	P-value
Araz, O.	1271	1.26 (0.9-1.62)	72.2%	0.003
In, E.	1603	1.22 (0.92-1.52)	72.8%	0.002
Bozkus, F.	1622	1.24 (0.83-1.65)	72.8%	0.003
Hilal, E.	1502	1.31 (1.03-1.59)	66.9%	0.010
Kostrubiec, M.	1519	1.28 (0.99-1.58)	70.8%	0.004
Akgullu, C.	1505	1.21 (0.91-1.52)	72.7%	0.003
Ertem, A	1447	1.15 (1.03-1.27)	0.6%	0.412

Supplementary Table 7. Risk factors for PE.

Risk factors	References
Age	[1]
Gender	[2, 3]
Race	[4, 5]
Thrombophilia	[6, 7]
Hospitalization	[8]
Surgery	[9]
Cancers	[10–12]
Obesity	[13, 14]
Estrogen	[15]
Prior history of thrombosis	[16, 17]
Central venous catheters	[18]
Cardiovascular disease	[19, 20]
Pregnancy	[21]
Immunological disease	[22–24]
Diabetes	[25]

Abbreviations: PE (Pulmonary embolism), MPV (Mean platelet volume), SD (Standard deviation), CI (Confidence intervals), EDTA (Ethylenediaminete traacetic acid), NOS (Newcastle–Ottawa Scale), WMD (Weighted mean differences), I^2 (I-square).

SUPPLEMENTARY REFERENCES

1. Righini M, Goehring C, Bounameaux H, Perrier A. Effects of age on the performance of common diagnostic tests for pulmonary embolism. Am J Med. 2000; 109:357–61. https://doi.org/10.1016/s0002-9343(00)00493-9 PMID:11020391

2. Tanabe Y, Yamamoto T, Murata T, Mabuchi K, Hara N, Mizuno A, Nozato T, Hisatake S, Obayashi T, Takayama M, Nagao K. Gender Differences Among Patients With Acute Pulmonary Embolism. Am J Cardiol. 2018; 122:1079–84. https://doi.org/10.1016/j.amjcard.2018.05.042 PMID:30072127

3. Jarman AF, Mumma BE, Singh KS, Nowadly CD, Maughan BC. Crucial considerations: Sex differences in the epidemiology, diagnosis, treatment, and outcomes of acute pulmonary embolism in non-pregnant adult patients. J Am Coll Emerg Physicians Open. 2021; 2:e12378. https://doi.org/10.1002/emp2.12378 PMID:33532761

4. White RH, Keenan CR. Effects of race and ethnicity on the incidence of venous thromboembolism. Thromb Res. 2009 (Suppl 4); 123:S11–17. https://doi.org/10.1016/S0049-3848(09)70136-7 PMID:19303496

5. Keenan CR, White RH. The effects of race/ethnicity and sex on the risk of venous thromboembolism. Curr Opin Pulm Med. 2007; 13:377–83. https://doi.org/10.1097/MCP.0b013e3281eb8ef0 PMID:17940480

6. Lian TY, Lu D, Yan XX, Tan JS, Peng FH, Zhu YJ, Wei YP, Wu T, Sun K, Jiang X, Hua L, Jing ZC. Association between congenital thrombophilia and outcomes in pulmonary embolism patients. Blood Adv. 2020; 4:5958–65. https://doi.org/10.1182/bloodadvances.2020002955 PMID:33275768

7. Obaid M, El-Menyar A, Asim M, Al-Thani H. Prevalence and Outcomes of Thrombophilia in Patients with Acute Pulmonary Embolism. Vasc Health Risk Manag. 2020; 16:75–85. https://doi.org/10.2147/VHRM.S241649 PMID:32214817

8. Aujesky D, Roy PM, Verschuren F, Righini M, Osterwalder J, Egloff M, Renaud B, Verhamme P, Stone RA, Legall C, Sanchez O, Pugh NA, N'gako A, et al. Outpatient versus inpatient treatment for patients with acute pulmonary embolism: an international,

open-label, randomised, non-inferiority trial. Lancet. 2011; 378:41–48.
https://doi.org/10.1016/S0140-6736(11)60824-6
PMID:21703676

9. Venclauskas L, Llau JV, Jenny JY, Kjaersgaard-Andersen P, Jans Ø, and ESA VTE Guidelines Task Force. European guidelines on perioperative venous thromboembolism prophylaxis: Day surgery and fast-track surgery. Eur J Anaesthesiol. 2018; 35:134–38.
https://doi.org/10.1097/EJA.0000000000000706
PMID:29112544

10. Ma L, Wen Z. Risk factors and prognosis of pulmonary embolism in patients with lung cancer. Medicine (Baltimore). 2017; 96:e6638.
https://doi.org/10.1097/MD.0000000000006638
PMID:28422863

11. Kraaijpoel N, Bleker SM, Meyer G, Mahé I, Muñoz A, Bertoletti L, Bartels-Rutten A, Beyer-Westendorf J, Porreca E, Boulon C, van Es N, Iosub DI, Couturaud F, et al, and UPE investigators. Treatment and Long-Term Clinical Outcomes of Incidental Pulmonary Embolism in Patients With Cancer: An International Prospective Cohort Study. J Clin Oncol. 2019; 37:1713–20.
https://doi.org/10.1200/JCO.18.01977 PMID:31116676

12. Silva P, Rosales M, Milheiro MJ, Santos LL. Pulmonary Embolism in Ambulatory Oncologic Patients. Acta Med Port. 2015; 28:463–68.
PMID:26574981

13. Sloan M, Sheth N, Lee GC. Is Obesity Associated With Increased Risk of Deep Vein Thrombosis or Pulmonary Embolism After Hip and Knee Arthroplasty? A Large Database Study. Clin Orthop Relat Res. 2019; 477: 523–32.
https://doi.org/10.1097/CORR.0000000000000615
PMID:30624321

14. Movahed MR, Khoubyari R, Hashemzadeh M, Hashemzadeh M. Obesity is strongly and independently associated with a higher prevalence of pulmonary embolism. Respir Investig. 2019; 57: 376–79.
https://doi.org/10.1016/j.resinv.2019.01.003
PMID:30770232

15. Weill A, Dalichampt M, Raguideau F, Ricordeau P, Blotière PO, Rudant J, Alla F, Zureik M. Low dose oestrogen combined oral contraception and risk of pulmonary embolism, stroke, and myocardial infarction in five million French women: cohort study. BMJ. 2016; 353:i2002.
https://doi.org/10.1136/bmj.i2002 PMID:27164970

16. Kim SH, Patel N, Thapar K, Pandurangadu AV, Bahl A. Isolated proximal greater saphenous vein thrombosis and the risk of propagation to deep vein thrombosis and pulmonary embolism. Vasc Health Risk Manag. 2018; 14:129–35.
https://doi.org/10.2147/VHRM.S164190
PMID:29928127

17. Hwang HG, Choi WI, Lee B, Lee CW. Incidence and Risk Factors of Recurrent Venous Thromboembolism after Pulmonary Embolism. Tuberc Respir Dis (Seoul). 2019; 82:341–47.
https://doi.org/10.4046/trd.2019.0019
PMID:31172708

18. Al-Asadi O, Almusarhed M, Eldeeb H. Predictive risk factors of venous thromboembolism (VTE) associated with peripherally inserted central catheters (PICC) in ambulant solid cancer patients: retrospective single Centre cohort study. Thromb J. 2019; 17:2.
https://doi.org/10.1186/s12959-019-0191-y
PMID:30697126

19. Protopapas AD, Baig K, Mukherjee D, Athanasiou T. Pulmonary embolism following coronary artery bypass grafting. J Card Surg. 2011; 26:181–88.
https://doi.org/10.1111/j.1540-8191.2010.01195.x
PMID:21320163

20. Pongmoragot J, Rabinstein AA, Nilanont Y, Swartz RH, Zhou L, Saposnik G, and Investigators of Registry of Canadian Stroke Network (RCSN) and University of Toronto Stroke Program for Stroke Outcomes Research Canada (SORCan [www.sorcan.ca]) Working Group. Pulmonary embolism in ischemic stroke: clinical presentation, risk factors, and outcome. J Am Heart Assoc. 2013; 2:e000372.
https://doi.org/10.1161/JAHA.113.000372
PMID:24275627

21. Dado CD, Levinson AT, Bourjeily G. Pregnancy and Pulmonary Embolism. Clin Chest Med. 2018; 39: 525–37.
https://doi.org/10.1016/j.ccm.2018.04.007
PMID:30122177

22. You H, Zhao J, Wang Q, Tian X, Li M, Zeng X. Characteristics and risk factors of pulmonary embolism in patients with systemic lupus erythematosus: a case control study. Clin Exp Rheumatol. 2020; 38:940–48.
PMID:31969219

23. Kridin K, Kridin M, Amber KT, Shalom G, Comaneshter D, Batat E, Cohen AD. The Risk of Pulmonary Embolism in Patients With Pemphigus: A Population-Based Large-Scale Longitudinal Study. Front Immunol. 2019; 10:1559.
https://doi.org/10.3389/fimmu.2019.01559
PMID:31396203

24. Chung WS, Lin CL, Hsu WH, Kao CH. Inflammatory bowel disease increases the risks of deep vein

thrombosis and pulmonary embolism in the hospitalized patients: a nationwide cohort study. Thromb Res. 2015; 135:492–96.
https://doi.org/10.1016/j.thromres.2014.12.025
PMID:25596768

25. Chung WS, Lin CL, Kao CH. Diabetes increases the risk of deep-vein thrombosis and pulmonary embolism. A population-based cohort study. Thromb Haemost. 2015; 114:812–18.
https://doi.org/10.1160/TH14-10-0868 PMID:26271946
